# The β-catenin/TCF-4-LINC01278-miR-1258-Smad2/3 axis promotes hepatocellular carcinoma metastasis

**DOI:** 10.1038/s41388-020-1307-3

**Published:** 2020-05-05

**Authors:** Wei-Juan Huang, Xiao-Peng Tian, Si-Xue Bi, Si-Rui Zhang, Ting-Sha He, Li-Yan Song, Jing-Ping Yun, Zhong-Guo Zhou, Rong-Min Yu, Mei Li

**Affiliations:** 1 Department of Pharmacology, College of Pharmacy, Jinan University, Guangzhou, China; 2 Integrated Chinese and Western Medicine Postdoctoral research station, Jinan University, Guangzhou, China; 3 Biotechnological Institute of Chinese Materia Medical, Jinan University, Guangzhou, China; 4 Sun Yat-sen University Cancer Center, State Key Laboratory of Oncology in South China, Collaborative Innovation Center of Cancer Medicine, Guangzhou, China; 5 Department of Pathology, Sun Yat-sen University Cancer Center, Guangzhou, China; 6 Department of Hepatology, Sun Yat-sen University Cancer Center, Guangzhou, China

**Keywords:** Liver cancer, Non-coding RNAs

## Abstract

Hepatocellular carcinoma (HCC) metastasis is largely responsible for HCC-associated recurrence and mortality. We aimed to identify metastasis-related long non-coding RNAs (lncRNAs) to understand the molecular mechanism of HCC metastasis. We first identified that miR-1258 was downregulated in HCC tissues both in The Cancer Genome Atlas (TCGA) and Sun Yat-sen University Cancer Center (SYSUCC) dataset. MiR-1258 expression negatively correlated with recurrence-free survival and overall survival of HCC patients. MiR-1258 overexpression inhibited migration and invasion of HCC cells both in vitro and in vivo, whereas miR-1258 downregulation promoted cell metastasis. Luciferase assays verified direct binding of miR-1258 to Smad2 and Smad3, thereby attenuating TGF-β/Smad signaling. We further established that lncRNA LINC01278 was a negative regulator of miR-1258. In vivo and in vitro assays demonstrated that LINC01278-mediated HCC metastasis was dependent on miR-1258 expression. Furthermore, miR-1258 downregulation in turn increased LINC01278 expression. We also observed that TCF-4 could bind to the LINC01278 promoter site. In addition, LINC01278 downregulation decreased migration and invasion of HCC cells induced by β-catenin and TGF-β1 both in vitro and in vivo. We uncovered a novel mechanism for β-catenin/TCF-4-LINC01278-miR-1258-Smad2/3 feedback loop activation in HCC metastasis, and the study indicated that LINC01278 could serve as a therapeutic target for HCC metastasis.

## Introduction

Hepatocellular carcinoma (HCC) is one of the most common malignancies worldwide, with at least 250,000 new cases every year, nearly half of them in China [1]. Although the treatment of HCC, especially early stage HCC, has greatly improved the survival of HCC patients, one half of patients with early stage HCC will develop recurrence after surgery [2–4]. The formation of metastases is largely responsible for HCC-specific recurrence and mortality [3, 5]. Therefore, it is important to understand the molecular mechanism in HCC metastasis in order to identify novel effective therapeutic targets.

Non-coding RNAs (ncRNAs) participate in various pathophysiological activities, especially in tumor development and progression [6]. Although the functions and mechanisms of microRNAs (miRNA) have been widely elaborated, the role of long non-coding RNAs (lncRNAs) in human cancers remains largely unknown. Genes can be positively or negatively regulated by lncRNAs through a diversity of mechanisms, and identification of cancer-related lncRNAs remains challenging [7]. Competitive endogenous RNA (ceRNA) hypothesis is one of the many hypotheses to explain the mechanism of lncRNAs [8]. This hypothesis proposed that lncRNAs competitively bind with miRNAs and harbor miRNA-response elements, thereby relieving the inhibitory effects of miRNA on the target mRNAs [8]. The “lncRNA–miRNA–mRNA” network has been validated in many human cancers [9, 10].

The function of these networks in HCC metastasis remains poorly understood. The activation of TGF-β/Smad and Wnt/β-catenin signaling plays an important role in metastasis of early stage HCC [11, 12]. It has been reported that phospho-Smad2/3 could stabilize β-catenin in the cytoplasm and facilitate translocation of β-catenin into the nucleus [13, 14]. However, very few studies documented that lncRNAs mediated the cross talk between the TGF-β1/Smad pathway and the Wnt/β-catenin signaling pathway [15].

In the current study, we aimed to identify metastasis-related lncRNAs. We discovered that the “LINC01278-miR-1258-Smad2/3” axis promotes HCC metastasis. More importantly, LINC01278 could be regulated by the Wnt/β-catenin signaling pathway. The feedback loop of “β-catenin/TCF-4- LINC01278-miR-1258-Smad2/3” may provide novel sight into HCC metastasis, and LINC01278 could serve as new therapeutic target.

## Results

### MiR-1258 expression negatively correlates with survival of HCC patients

**Fig. 1:**
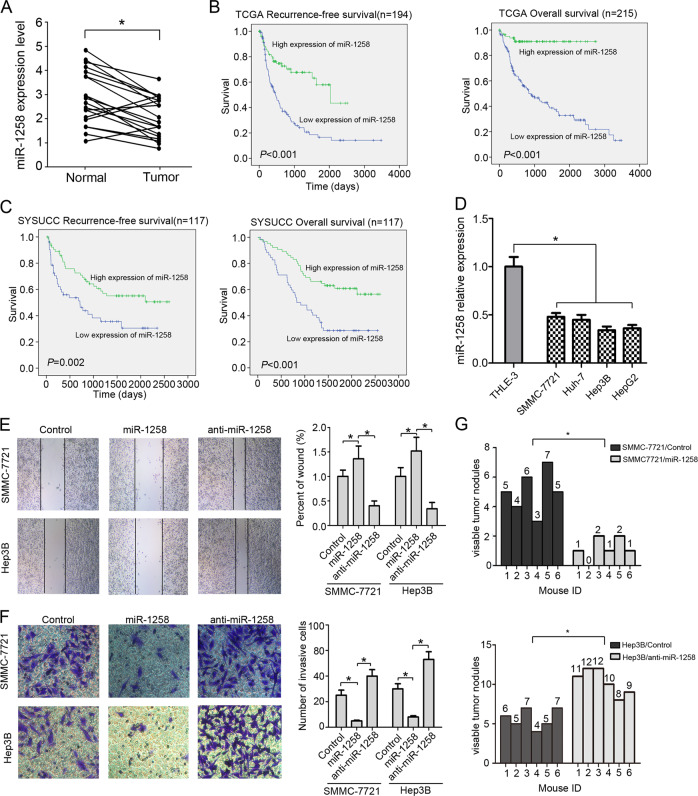
miR-1258 suppresses HCC cell metastasis in vitro and in vivo and its expression associated with E-HCC recurrence and patient’s survival. **a** The expression of miR-1258 in 20 paired E-HCC tissues and adjacent normal tissues. **b** Correlation of recurrence-free survival (RFS) and overall survival (OS) and miR-1258 expression by Kaplan–Meier analysis in TCGA datasets. **c** Correlation of RFS and OS and miR-1258 expression by Kaplan–Meier analysis in SYSUCC datasets. **d** The expression of miR-1258 in immortalized liver cells (THLE-3) and four HCC cell lines (SMMC-7721, Huh-7, HepG2, and Hep3B). **e** The wound-healing assay of HCC cells. **f** The invasion assay of HCC cells. **g** The numbers of lung metastasis. Experiments performed in triplications. miR-1258, HCC cells were transfected by miR-1258 expression vector. Anti-miR-1258, HCC cells were transfected by miR-1258 antisense plasmid. *P < 0.05.

We identified 29 differentially expressed miRNAs between 49 normal tissues and 369 HCC tissues in the TCGA dataset using the significant analysis of microarray (SAM) algorithm, including 25 upregulated miRNAs and 4 downregulated miRNAs (Table 1). MiRNA-1258 was the top downregulated miRNA (fold change = 0.32, *P* < 0.001). Our ROC analysis showed that the optimal cutoff of miR-1258 expression in TCGA dataset was 1.226 (Fig. S1) and was used categorized the study patients into low versus high miR-1258 expression group. The clinicopathologic characteristics of 117 patients from the SYSUCC dataset stratified by low versus high expression of miR-1258 and LINC01278 are summarized in Table S1. We next sought to validate the differentially expressed miRNAs in 20 paired early stage HCC and adjacent normal tissues from the SYSUCC dataset. We found that HCC tissues had significantly lower levels of miR-1258, miR-1248, miR-369, and miR-7704 than paired adjacent normal tissues (Fig. 1a and S2).

**Table 1:** Identification of differential miRNAs between 49 normal liver tissues and 369 HCC tissues in the TCGA liver cancer dataset using significant analysis of microarrays (SAM).

Gene ID	MicroRNAs	Fold change (HCC tissue versus normal tissue)	*P* value
MIMAT0004764	miR-490	3.086741	0.00046
MIMAT0019773	miR-4686	2.963208	0.00046
MIMAT0000428	miR-135a	2.931293	0.00046
MIMAT0000095	miR-96	2.703935	0.00046
MIMAT0004556	miR-10b	2.613535	0.00046
MIMAT0005798	miR-1185	2.490991	0.00046
MIMAT0004952	miR-665	2.486725	0.00046
MIMAT0000430	miR-138	2.470451	0.00046
MIMAT0019880	miR-4746	2.36657	0.00046
MIMAT0011156	miR-2114	2.300792	0.000504
MIMAT0002823	miR-512	2.188039	0.00549
MIMAT0003215	miR-552	2.179489	0.001036
MIMAT0018198	miR-3923	2.139169	0.004226
MIMAT0000454	miR-184	2.127808	0.000547
MIMAT0026615	miR-552	2.11621	0.000618
MIMAT0019978	miR-4800	2.115838	0.00046
MIMAT0030021	miR-7706	2.109118	0.00046
MIMAT0022844	miR-216a	2.097376	0.000464
MIMAT0019071	miR-4532	2.09168	0.00046
MIMAT0030425	miR-7850	2.071784	0.000461
MIMAT0022494	miR-5701	2.037641	0.00046
MIMAT0005924	miR-1270	2.031196	0.00047
MIMAT0019979	miR-4800	2.013864	0.00046
MIMAT0004185	miR-820	2.01187	0.00046
MIMAT0015085	miR-3200	2.010477	0.000463
MIMAT0005900	miR-1248	0.480085	0.00046
MIMAT0001621	miR-369	0.463557	0.00046
MIMAT0030019	miR-7704	0.360133	0.00046
MIMAT0005909	miR-1258	0.323214	0.00046

We next analyzed correlation between miR-1258 expression and recurrence-free survival (RFS) and overall survival (OS) of HCC patients in the TCGA dataset. Kaplan–Meier analyses showed that HCC patients with high miR-1258 expression had significantly better median RFS and OS than those with low miR-1258 expression (*P* < 0.001) (Fig. 1b). Significant difference in RFS (*P* = 0.002) and OS (*P* < 0.001) was also observed between the low and high miR-1258 expression group in the SYSUCC dataset (Fig. 1c).

### MiR-1258 inhibits in vitro and in vivo migration of HCC cells

We further investigated the effects of miR-1258 on the migration of HCC cells in vitro and in vivo. miR-1258 expression was significantly lower in four HCC cell lines SMMC-7721, Huh-7, HepG2, and Hep3B versus normal liver cell line THLE-3 (Fig. 1d). We infected SMMC-7721 and Hep3B cells with lentiviruses overexpressing miR-1258 or antisense miR-1258 (Fig. S3). The wound scratch assays and transwell invasion assays demonstrated that ectopically expressed miR-1258 significantly suppressed, whereas miR-1258 downregulation marked enhanced the migration and invasion of HCC cells (Fig. 1e, f). Furthermore, mice bearing SMMC-7721 xenografts overexpressing miR-1258 had a significantly lower number of metastatic lung nodules than the control mice. Meanwhile, mice bearing Hep3B xenografts expressing antisense miR-1258 had a significantly higher number of metastatic lung nodules than the control mice (Fig. 1g and S4).

### MiR-1258 targets Smad2/3 in HCC

**Fig. 2:**
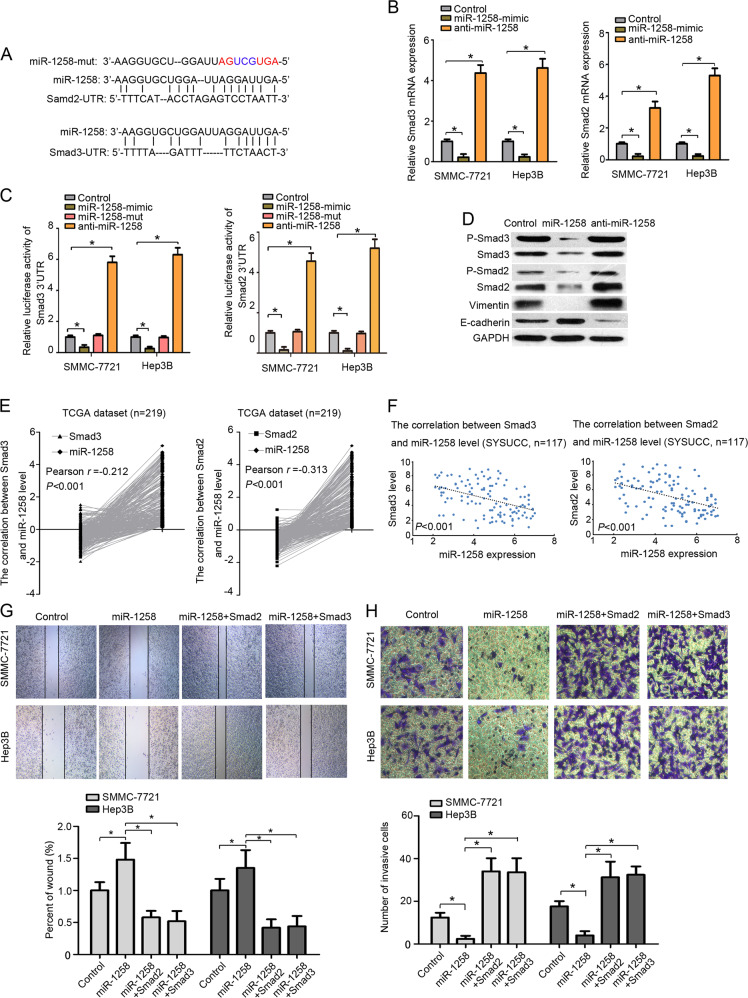
MiR-1258 target Smad2/3 in HCC cells. **a** The predicted target sequence of miR-1258 in 3′UTR of Smad2 (Smad2-3′UTR) and Smad3 (Smad3-3′UTR) and mutant containing three altered nucleotides in the seed sequence of miR-1258 (miR-1258-mut). **b** Luciferase assay of pGL3-Smad2-3′UTR and pGL3-Smad3-3′UTR in the presence of miR-1258 mimics, miR-1258-mutant, and anti-miR-1258. **c** The Smad2/3 mRNA levels after transfected by miR-1258 mimics, miR-1258-mutant, and anti-miR-1258. **d** The protein levels of P-Smad2, Smad2, P-Smad3, Smad3, PAI-1, E-cadherin, and vimentin after transfected by miR-1258 mimics and anti-miR-1258 in HCC cell. **e** The correlation between Smad2/3 gene expression and miR-1258 level in TCGA dataset. **f** The correlation between Smad2/3 gene expression and miR-1258 level in SYSUCC dataset. **g**, **h** The wound-healing assay and transwell assay of HCC cells transfected with miR-1258, miR-1258+Smad2, miR-1258+Smad3. Experiments performed in triplications. miR-1258, HCC cells were transfected by miR-1258 expression vector. Anti-miR-1258, HCC cells were transfected by miR-1258 antisense plasmid. miR-1258+Smad2, co-overexpressed miR-1258, and Samd2 in HCC cells. miR-1258+Smad3, co-overexpressed miR-1258, and Samd3 in HCC cells. *P < 0.05.

An online miRNA target prediction database (*miRecords*) showed that both *Smad2* and *Smad3*, two well-known positive regulators of tumor metastasis, contained putative binding sites for miR-1258 (Fig. 2a). Quantitative RT-PCR assays revealed that miR-1258-mimics significantly increased while anti-miR-1258 markedly decreased the mRNA levels of *Smad2* and *Smad3* in HCC cells (Fig. 2b). The luciferase reporter assays further showed that miR-1258 mimics significantly suppressed while antisense miR-1258 markedly enhanced the luciferase activities of Smad2-3′UTR (luc-Smad2-3′UTR) and Smad3-3′UTR (luc-Smad3-3′UTR). Meanwhile, miR-1258-mut had no effect on the luciferase activities of luc-Smad2-3′UTR and luc-Smad3-3′UTR (Fig. 2c). Furthermore, our immunoblotting assays showed that ectopically expressed miR-1258 reduced while antisense miR-1258 increased Smad2 and Smad3 levels. We next investigated the effect of miR-1258 on *E*-cadherin, an epithelial–mesenchymal transition marker. We found that miR-1258 mimics apparently enhanced while antisense miR-1258 markedly suppressed *E*-cadherin expression in HCC cells. In contrast, miR-1258 mimics significantly suppressed while antisense miR-1258 markedly enhanced the expression of vimentin (Fig. 2d).

Pearson correlation analysis of 269 HCC samples from the TCGA dataset demonstrated significant negative correlation between both Smad2 and Smad3 mRNA transcript levels and miR-1258 expression (Smad3: *r* = −0.212, *P* < 0.001; Smad2: *r* = −0.313, *P* < 0.001) (Fig. 2e). A significant negative correlation between both Smad2 and Smad3 mRNA transcript levels and miR-1258 expression was also observed in 117 early stage HCC samples from the SYSUCC dataset (Fig. 2f). Wound scratch assays and transwell assays further showed that restoration of Smad2 and Smad3 expression significantly promoted the migration and invasion of HCC cells overexpressing miR-1258 (Fig. 2g, h).

### MiR-1258 is regulated by LINC01278

**Fig. 3:**
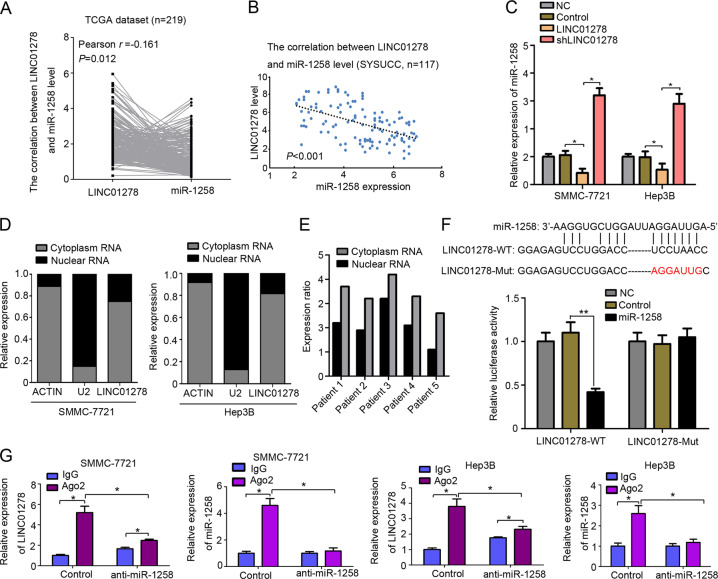
MiR-1258 was regulated by LINC01278. **a** The correlation between LINC01278 gene expression and miR-1258 level in TCGA dataset. **b** The correlation between LINC01278 gene expression and miR-1258 level in SYSUCC dataset. **c** The expression of miR-1258 in HCC cells transfected by LINC01278 and shLINC01278. **d** LINC01278 is abundant in cytoplasm of SMMC-7721 and Hep3B cells. U2 and actin were used as positive control. **e** Cytoplasm enrichment of LINC01278 in HCC patients’ tissues. **f** The predicted binding sites between miR-1258 and LINC01278. The muted site (LINC01278-mut) was used for luciferase reporter assay. The relative luciferase activities were detected in 293 T cells transfected by LINC01278-WT and LINC01278-mut. **g** RNA-IP was used to identify the inhibition of miR-1258 by LINC01278. The expression levels of LINC01278 and miR-1258 were detected using qRT-PCR. LINC01278, ectopic LINC01278 expression in HCC cells. shLINC01278, HCC cells were transfected by shRNA target LINC01278. *P < 0.05.

LncRNAs have been increasingly shown to correlate with miRNA expression regulation. Therefore, the bioinformatic tool LncBase v.2 was used to identify candidate lncRNAs capable of regulating miR-1258 (Table S2). Pearson correlation analysis of the TCGA dataset demonstrated a significant negative correlation between miR-1258 expression and LINC01278 among the top ten predicted lncRNAs (Fig. 3a), which was also observed in 117 early stage HCC samples from the SYSUCC dataset (Fig. 3b). Moreover, LINC01278 significantly reduced while shRNAs against LINC01278 markedly elevated miR-1278 levels (Fig. 3c). Subcellular fractionation analysis revealed that LINC01278 was mainly cytoplasmic both in HCC cells and HCC tissues (Fig. 3d, e). Our dual-luciferase gene reporter assays further showed that co-transfection of LINC01278-wt and miR-1258 significantly decreased luciferase activities while co-transfection of LINC01278-mut and miR-1258 had no effect on luciferase activities compared with the control group (Fig. 3f).

RNA immunoprecipitations were undertaken to understand the mechanism whereby LINC01278 regulates miR-1258. In the control group, the amount of LINC01278 and miR-1258 that immunoprecipitated with Ago2 was higher than the IgG group. In the anti-miR-1258 group, the amount of LINC01278 and miR-1258 that immunoprecipitated with Ago2 was significantly decreased compared with the control group (Fig. 3g). The results suggested that LINC01278 regulated miR-1258 expression by mediating with RNA-induced silencing complex (RISC). Notably, the amount of LINC01278 was higher than the IgG group in the anti-miR-1258 group, implying that miR-1258 may have an inhibitory feedback in LINC01278 expression (Fig. 3g).

### LINC01278 upregulates Smad2 and Smad3 and promotes HCC metastasis in vivo and in vitro

**Fig. 4:**
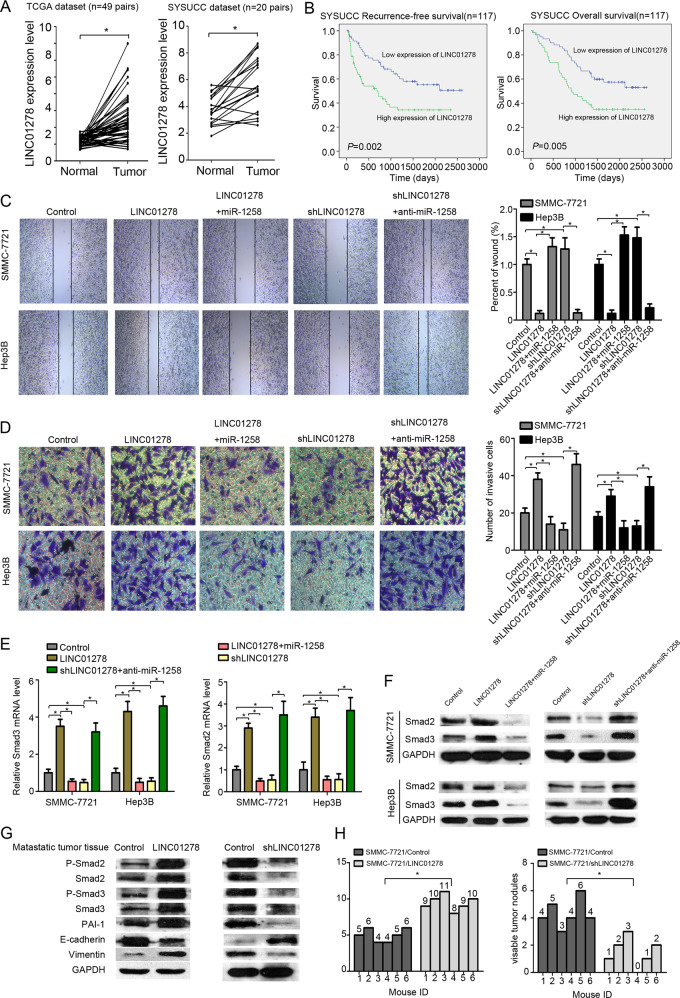
LINC01278 increases HCC metastasis in vivo and in vitro. **a** The expression of LINC01278 in paired HCC tissues and adjacent normal tissues in TCGA datasets (*n* = 49) and SYSUCC dataset (*n* = 20). **b** Correlation of recurrence-free survival (RFS) and overall survival (OS) and LINC01278 expression by Kaplan–Meier analysis in SYSUCC dataset. **c** The wound-healing assay in HCC cells. **d** The invasion assay in HCC cells. **e** The Smad2/3 mRNA expression of HCC cells transfected by LINC01278, shLINC01278, LINC01278+miR-1258, and shLINC01278+anti-miRNA-1258. **f** The protein expression of Smad2 and Smad3 in HCC cells transfected by LINC01278, shLINC01278, LINC01278+miR-1258, and shLINC01278+anti-miRNA-1258. **g** The protein levels of P-Smad2, Smad2, P-Smad3, Smad3, PAI-1, E-cadherin, and vimentin in metastasis tumor tissues. LINC01278, ectopic LINC01278 expression in HCC cells. **h** The lung metastasis of HCC cells transfected by LINC01278 and shLINC01278. shLINC01278, HCC cells were transfected by shRNA target LINC01278. miR-1258, HCC cells were transfected by miR-1258 expression vector. Anti-miR-1258, HCC cells were transfected by miR-1258 antisense plasmid. *P < 0.05.

We next measured LINC01278 expression in paired HCC and adjacent normal tissues from the TCGA dataset and the SYSUCC dataset. Quantitative RT-PCR assays showed significantly higher LINC01278 expression in HCC tissues versus paired adjacent normal tissues in both the TCGA dataset (*n* = 49 pairs) and the SYSUCC dataset (*n* = 20 pairs) (*P* < 0.05) (Fig. 4a). Using median LINC01278 expression as a cutoff for the SYSUCC cohort, patients with high LINC01278 expression had significantly worse RFS and OS than patients with low LINC01278 expression (Fig. 4b). Wound scratch assays and transwell assays showed that LINC01278 overexpression significantly promoted the migration and invasion of HCC cells, which, however, was attenuated by ectopically expressed miR-1258 (Fig. S5 and 4c, d). In addition, LINC01278 downregulation by shRNAs against LINC01278 markedly suppressed the migration and invasion of HCC cells, which was alleviated by miR-1258 downregulation (Fig. 4c, d).

We further investigated the effect of LINC01278 on the expression of Smad2 and Smad3, which are the targets of miR-1258. We observed that LINC01278 overexpression significantly increased while shRNAs against LINC01278 markedly depressed the protein and mRNA levels of Smad2 and Smad3 in HCC cells (Fig. 4e, f). MiR-1258 upregulation in HCC cells overexpressing LINC01278 markedly reduced while miR-1258 downregulation significantly increased the protein and mRNA transcript levels of Smad2 and Smad3 (Fig. 4e, f). In HCC tissues metastasized to the lungs, LINC01278 overexpression increased the protein levels of phospho-Smad2, Smad2, phospho-Smad3, Smad3, PAI-1, and vimentin and reduced the levels of *E*-cadherin (Fig. 4g). Meanwhile, shRNAs against LINC1278 decreased the protein levels of phospho-Smad2, Smad2, phospho-Smad3, Smad3, PAI-1, and vimentin and increased the levels of *E*-cadherin. Furthermore, in the HCC tissues metastasized to the lungs, LINC01278 overexpression significantly increased while shRNAs against LINC1278 markedly reduced the number of metastatic lung nodules (Fig. 4h and S6).

### LINC01278 is a mediator between the Wnt/β-catenin and TGF-β/Smad signaling pathways

**Fig. 5:**
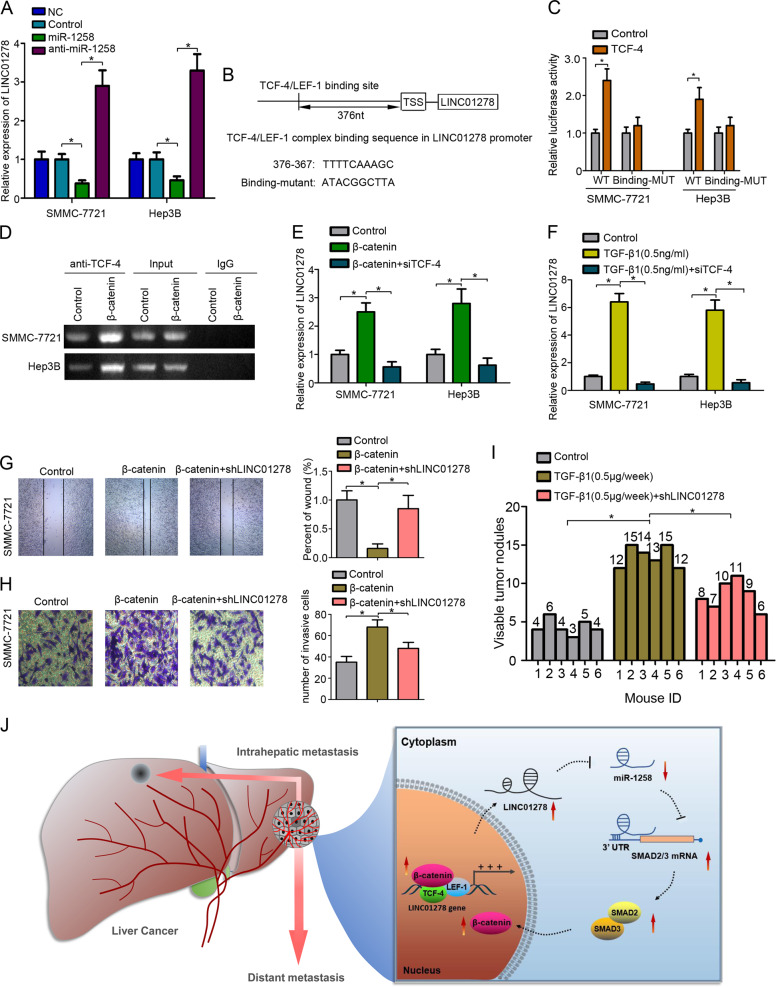
LINC01278 was regulated by TCF-4. **a** The expression of LINC01278 in HCC cells transfected by miR-1258 and anti-miR-1258. **b** The TCF-4/LEF-1 binding site in the LINC01278’s promoter sequence was identified. The mutant sequence was designed for the luciferase reporter assay. **c** The relative luciferase activity was detected in 293 T cells co-transfected by binding-WT/binding-mut and TCF-4. **d** ChIP assay was used to detect the TCF-4 to the TRE (TCF responsive element: TTCAAAG) regions in LINC01278 promoter. **e** The relative expression of LINC01278 in HCC cells transfected by β-catenin and siTCF-4. **f** The relative expression of LINC01278 in HCC cells treated with TGF-β1 (0.5 ng/ml) and siTCF-4. **g**, **h** The wound-healing assay and invasion assay in SMMC-7721 cells. **i** The lung metastasis of SMMC-7721 cells treated with TGF-β1 (0.1 µg/each time, three times a week) and shLINC01278. **j** Schematic illustration of “β-catenin/TCF-4-LINC01278-miR-1258-Smad2/3” axis. miR-1258, HCC cells were transfected by miR-1258 expression vector. Anti-miR-1258, HCC cells were transfected by miR-1258 antisense plasmid. TCF-4, HCC cells were transfected by TCF-4 expression vector. β-catenin, HCC cells were transfected by β-catenin expression vector. siTCF-4, HCC cells were transfected by siRNA target TCF-4. shLINC01278, HCC cells were transfected by shRNA target LINC01278. *P < 0.05.

The RNA immunoprecipitation results prompted us to speculate that miR-1258 may have an inhibitory feedback on LINC01278 expression. Our RT-PCR assays showed that ectopically expressed miR-1258 significantly depressed LINC01278 expression (Fig. 5a). Analysis of the promoter sequence of LINC01278 using the online PROMO algorithm identified a putative TCF-4/LEF-1 binding site (Fig. 5b). Luciferase reporter assays further revealed that HCC cells co-transfected with LINC01278-wt and TCF-4 showed significantly increased luciferase activities relative to the control group, whereas HCC cells co-transfected with TCF-4 and LINC01278 with the binding sequence mutated showed no significant change in luciferase activities versus the control group (Fig. 5c). The mRNA levels of LINC01278 were significantly decreased by suppressing the expression of β-catenin, TCF-4, and Smad2/3 (Fig. S7). Chromatin immunoprecipitation (ChIP) assays further showed that TCF-4 bound to the LINC01278 promoter. Moreover, TCF-4 binding to LINC01278 promoter increased upon β-catenin overexpression (Fig. 5d). In addition, β-catenin overexpression caused a significant increase in LINC01278 expression in HCC cells, which, however, was aborted by co-transfection with siTCF-4 (Fig. 5e). Furthermore, TGF-β1 (0.5 ng/ml) upregulated while co-transfection with siTCF-4 diminished LINC01278 expression (Fig. 5f). The expression of signaling molecules of β-catenin/TCF-4-LINC01278-miR-1258-Smad2/3 feedback loop was listed in Fig. S7.

We further evaluate whether LINC01278 could be as a therapeutic target for metastatic HCC. Wound scratch assays and transwell assays showed that ectopically expressed β-catenin promoted the migration and invasion of HCC cells, which was aborted by shRNAs against LINC01278 (Fig. 5g, h). In addition, TGF-β1 (0.5 μg/week) significantly increased the number of metastatic lung nodules while TGF-β1 plus shLINC01278 reduce the number of metastatic lung nodules (Fig. 5i, and S8).

## Discussion

In this study, we show that the “β-catenin/TCF-4-LINC01278-miR-1258-Smad2/3” axis plays an important role in regulating HCC metastasis (Fig. 5j), and LINC01278 may provide a potential therapeutic strategy for preventing HCC metastasis.

To identify HCC metastasis-related lncRNAs, we first explored the TCGA dataset to identify differentially expressed miRNAs. Investigation of E-HCC’s metastasis is important in providing useful insight into treatment strategy, and we selected an E-HCC database from SYSUCC for validation. We found that miR-1258 was one of the most significantly decreased miRNAs both in the TCGA dataset and the SYSUCC dataset. Furthermore, miR-1258 was associated with HCC prognosis in both cohorts. In vitro assays demonstrated that miR-1258 expression negatively correlated with metastasis of HCC cells. MiR-1258 regulates cell cycle progression and inhibits cell proliferation, and suppresses tumor metastasis in many cancer types [16–18]. In this study, we screened the targets of miR-1258 by online tools. We noticed that both Smad2 and Smad3 were the putative targets of miR-1258. Luciferase reporter assays demonstrated miR-1258 binding to Smad2-3′-UTR and Smad3-3′-UTR, thereby inhibiting Samd2/3 expression. In the classic Smad pathway, TGF-β signaling activates Smad2 and Smad3 through a tetramer complex of type I and type II receptors and binds to Smad4 [19]. The Smad complex translocate to the nucleus to mediate inhibition or activation of target genes, thereby promoting epithelial–mesenchymal transition [20, 21]. MiR-1258 specifically targets Smad2 and Smad3, block the TGF-β/Smad signaling pathway, and inhibit metastasis of HCC cells.

The “ceRNA” hypothesis speculates that lncRNA could regulate mRNA expression by sponging miRNAs [22]. We used lncRNA predictive online tools to screen putative lncRNAs which could affect miR-1258 function. We identified LINC01278, located on chromosome Xq11.1, as a novel lncRNA that could directly target miR-1258. LINC01278 was downregulated in papillary thyroid carcinoma compared with normal tissues and acted as a tumor suppressor in papillary thyroid carcinom progression [23]. However, in this study, we found that the expression of LINC01278 was significantly increased in HCC tissues compared with adjacent normal tissues in the TCGA dataset and the SYSUCC dataset. Moreover, high LINC01278 expression was associated with worse prognosis in HCC. Both in vitro and in vivo assays demonstrated that LINC01278 promoted HCC cell metastasis dependent on miR-1258 expression. The results suggest that LINC01278 may play different roles in different tumors. Previous studies demonstrated that RISC, which is the key molecular for siRNA or miRNA-mediated gene silencing, participates in the “ceRNA” network, and Ago2 is the core component of RISC [24]. We conducted RNA immunoprecipitations to investigate the underlying mechanism whereby LINC01278 targets miR-1258. We found that the levels of LINC01278 and miR-1258 immunoprecipitated with Ago2 increased. MiR-1258 upregulation significantly increased the levels of LINC01278 immunoprecipitated with Ago2. The results suggested that LINC01278 could regulate miR-1258 by RISC complex, and other miRNAs could also bind to LINC01278.

We noticed that the levels of miR-1258 could reciprocally regulate the expression of LINC01278. We first speculated that the promoter of LINC01278 may have a Smad responsive element (SRE) [25]. We used the online PROMO algorithm to analyze putative factor binding sites. However, no SRE was present in the LINC01278 promoter. According to the online tool, transcription factors TCF-4 and LEF-1 bind to the promoter of LINC01278 which was confirmed by luciferase reporter assays and ChIP assays. Our quantitative RT-PCR analysis further showed that both β-catenin and TGF-β1 induced LINC01278 expression. LINC01278 downregulation could inhibit the HCC metastasis induced by β-catenin and TGF-β1. Many studies have shown that phospho-Smad2/3 complex could stabilize β-catenin in the cytoplasm, and promote translocation of β-catenin into the nucleus [13, 26]. In this study, we found that the “β-catenin/TCF-4-LINC01278-miR-1258-Smad2/3” axis could in turn induce the expression of LINC01278, forming a positive feedback loop. It should be noticed that the amount of TGF-β1 secreted by hepatic stellate cells was usually elevated under long-term chronic inflammation, such as alcohol or hepatitis virus [27]. The TCGA network also showed that Wnt signaling is largely altered in HCC [28]. Therefore, LINC01278, which is an important cross-mediator between TGF-β/Smad signaling and Wnt signaling, could serve as a new therapeutic target to inhibit HCC metastasis.

In conclusion, our results showed that the “LINC01278-miR-1258-Samd2/3” axis promotes HCC metastasis. The axis forms a positive feedback loop and increases the stability of TGF-β signaling and Wnt/β-catenin signaling, thereby promoting HCC metastasis. LINC01278 could serve as a new therapeutic target for HCC metastasis.

## Materials and methods

### Cells

Four HCC cell lines (SMMC-7721, Huh-7, HepG2, and Hep3B), and immortalized human liver cells (THLE-3) (China Center for Type Culture Collection, Wuhan, China; American Type Culture Collection, Manassas, VA, USA) were grown in Dulbecco’s modified Eagle’s medium (Invitrogen, Carlsbad, CA, USA) with 10% newborn calf serum at 37 °C in a humidified atmosphere containing 5% CO_2_. All cell lines were authenticated based on short tandem repeat fingerprinting.

### Acquisition of tissue specimens

Twenty paired HCC tissue and adjacent normal tissue specimens archived at Sun Yat-sen University Cancer Center (SYSUCC) between June 1, 2017 and July 30, 2018 were obtained. In addition, 117 early stage HCC (defined as diameters < 5 cm) samples surgically resected at SYSUCC between January 1, 2008 and December 30, 2013 were acquired. Major exclusion criteria were preoperative ablation, radiotherapy, transarterial chemoembolization, or liver transplant. All eligible patients had definitive diagnosis and follow-up data. Informed content was obtained from all subjects. The study protocol was approved by Institutional Review Board of SYSUCC.

### Analysis of online database

The clinical, miRNA and RNA-Seq data of patients in the Cancer Genome Atlas Liver Hepatocellular Carcinoma (TCGA-LIHC) data collection were downloaded from the TCGA database (The Cancer Genome Atlas Program) (https://portal.gdc.cancer.gov/). Online miRNA target prediction database (*miRecords*) (http://c1.accurascience.com/miRecords/) was used to identify potential miR-1258 target genes [29]. The bioinformatic tool LncBase v.2 (http://carolina.imis.athena-innovation.gr/diana_tools/web/index.php?r=site%2Findex) was used to identify candidate lncRNAs capable of regulating miR-1258 [30]. The online PROMO algorithm analysis (http://alggen.lsi.upc.es/cgi-bin/promo_v3/promo/promoinit.cgi?dirDB=TF_8.3) was used to identify the putative transcription factor binding with the promoter sequence of LINC01278 [31, 32].

### Wound scratch assays and transwell assay

For wound scratch assays, cells were cultured into a confluent monolayer. A P200 pipette tip was used to introduce a scratch, and the fraction of cell coverage was measured at 48 h. For invasion assays, 1 × 10^4^ cells were seeded into the upper Matrigel invasion chamber (BD Biosciences, NJ, USA) and FBS was added into the lower chamber. The invading cells were counted on the lower side of the chambers at 24 h in six randomly chosen microscopic fields per filter at ×200 magnification using an IX71 inverted microscope (Olympus Corp.). The assays were performed at least three times independently in triplicate.

### Cell fractionation assays

Cell fractionation assays were performed using the PARIS Kit (Life Technologies, Carlsbad, CA, USA) as instructed by the manufacturer. Actin was used as the positive marker for the cytoplasmic fraction and small nuclear RNA U2 was used as a marker for the nucleus.

### Quantitative real-time PCR

Total cellular RNA was extracted using TRIzol reagents (Invitrogen) and cDNA synthesis was done using the PrimeScript RT reagent Kit (Promega, Madison, WI, USA) as instructed by the manufacturer. Real-time PCR was done on an ABI 7900HT Fast Real-time PCR system (Applied Biosystems, Foster City, CA, USA). MiR-1258 expression was examined using Bulge-Loop^TM^ miRNA kit (RuiBo, Guangzhou, China) with U6 as a control. All the primers were purchased from Gene Copoeia Co. (Guangzhou, China). The sequence of primers is shown in Table S3.

### Western blot analysis

Cellular lysates were prepared using RIPA buffer. Cellular proteins (25 mg) were resolved by polyacrylamide-SDS gel electrophoresis and transferred to polyvinylidene difluorid membranes (Millipore) using a standard procedure. The membranes were incubated with mouse anti-P-Smad2 (1:1000, Abcam), mouse anti-Smad2 (1:1000, Abcam), mouse anti-P-Smad3 (1:1500, ProteinTech), mouse anti-Smad3 (1:1500, ProteinTech), rabbit anti-*E*-cadherin (1:1000, Abcam), rabbit anti-vimentin (1:1000, Abcam), and rabbit anti-PAI-1 (1:1000, Abcam) antibodies. Anti-rabbit or anti-mouse IgG was used as secondary antibody (1:8000). Densitometry was done using Quantity One 4.4.0 software.

### Lentiviral infection

pCDH-CMV-EF1-Puro plasmid was used for expression vector. MiR-1258 antisense plasmid, miR-1258 expression vector, miR-1258-mut, Smad2 expression vector, Smad3 expression vector, LINC01278 expression vector, LINC01278-mut vector, shLINC01278 vector, LINC01278-binding-mut vector, β-catenin expression vector, TCF-4 expression vector, siTCF-4, were purchased from Kangcheng Biotechnology Co (Guangzhou, China). The sequence of shLINC01278 and siTCF-4 is shown in Table S3. siSmad2 (sc-38374), siSmad3 (sc-38376), and siβ-catenin (sc-29209), were purchased from Santa Cruz. The sequence of miR-1258-mut and LINC01278-mut is shown in Figs. 2a and 3f. The pCDH-CMV-MCS-EF1, pCMV/pVSVG, pRSV/pREV, and pMDLG/pRRE were used to construct lentivirus particles. After cultured 48 h, the lentiviruses were produced in culture medium by 293FT cells by using Lipofectamine 2000 reagent (Invitrogen). The culture medium containing the lentiviruses was centrifuged at low temperature for 4 h. Then, HCC cells were infected with concentrated lentiviruses at a multiple of infection of 10 plus 8 mg/ml polybrene (Sigma, St Louis, MO, USA).

### RNA immunoprecipitation assays

RNA immunoprecipitation assays were performed using EZ-Magna RIP RNA-binding protein immunoprecipitation kit (Millipore) following the manufacturer’s instructions. The cellular lysates were incubated with magnetic beads conjugated with human anti-Argonaute2 (Ago2) antibody (Millipore). Mouse IgG (Millipore) served as the negative control. The immunoprecipitated RNA was isolated using proteinase K buffer and the target RNAs were analyzed by quantitative RT-PCR as detailed elsewhere [33].

### ChIP assays

The nuclear extract was obtained using the Nuclear Extraction Kit (Active Motif, California, USA) as instructed by the manufacturer. Then, immunoprecipitation of the nuclear lysates was performed using a commercially available ChIP assay kit (Abcam) according to the manufacturer’s protocol. Rabbit anti-TCF-4 antibody (1:50, Abcam) and nonspecific IgG (1:200, Sigma) were used for immunoprecipitation. The immunoprecipitated promoter fragment containing the TCF response element was amplified by PCR and visualized by agarose gel electrophoresis.

### Luciferase reporter assays

We cloned the 3′UTR of *Smad2* and *Smad3* (Gene Copoeia) to the downstream of the luciferase gene in pGL3-basic vector (Promega), and then transfected the miR-1258-mimic, miR-1258-mut, anti-miR-1258, and miR-control HCC cells with the modified vectors. The LINC01278 promoter and LINC01278-binding-nut promoter in which the TCF-4-binding site was mutated (Gene Copoeia) were cloned into pGL3-Basic vector (Promega), and the TCF-4 overexpression and control HCC cells were transfected with the modified vectors. The Renilla luciferase reporter pRL-TK was used as a control. LINC01278 gene and LINC01278-mut sequence (Gene, Copoeia) were cloned into pmirGLO vector (Promega). After 48 h, firefly and Renilla luciferase activities were measured using Dual-Luciferase Reporter Kit (Promega).

### HCC xenograft assays

The animal study was approved by the Animal Care and Use Committee of SYSUCC and carried out in strict accordance with the established institutional guidelines and the USA NIH guidelines on the use of experimental animals. Totally 5 × 10^6^ HCC cells were subcutaneously inoculated into the flanks of 4-week-male BALB/C-nu/nu athymic nude mice. After the subcutaneous tumors reached 1 mm^3^ in volume, they were implanted in the left hepatic lobe of nude mice. On day 50 post hepatic implantation, mice were sacrificed and lung metastatic nodules were enumerated by consecutive tissue sections as described [34].

### Statistical analysis

The miRNA expression profiles of HCC patients in the TCGA dataset were examined using the SAM algorithm and miRNA with a fold change ≥2 was considered differentially expressed. ROC analysis was used to determine optimal cutoff value of miR-1258 and LINC01278. Statistical analysis was performed using SPSS 17.0 software (SPSS, Chicago, IL, USA). RFS was calculated from the date of surgery to pathological confirmation of tumor recurrence of surgical. OS was calculated from the date of surgery to the date of death of any cause. Survival was censored at the date of the last follow-up visit. Survival curves were calculated by Kaplan–Meier method and compared by the log-rank test. Pearson correlation analysis was used to assess correlation between two variables. Chi-square test was used to assess correlation between miR-1258 and LINC01278 levels and clinicopathologic features. Data were presented as mean ± SD and assessed by Student’s *t* test. *P* values < 0.05 were considered statistically significant.

## Supplementary information

Figure S1

Figure S2

Figure S3

Figure S4

Figure S5

Figure S6

Figure S7

Figure S8

Table S1

Table S2

Table S3

Supplementary Figure Legends
