# Supplementary figures and images for: The β-catenin/TCF-4-LINC01278-miR-1258-Smad2/3 axis promotes hepatocellular carcinoma metastasis
Source: Oncogene. 2020 May 5;39(23):4538–50. doi: 10.1038/s41388-020-1307-3 (PMC7269911; doi:10.1038/s41388-020-1307-3)

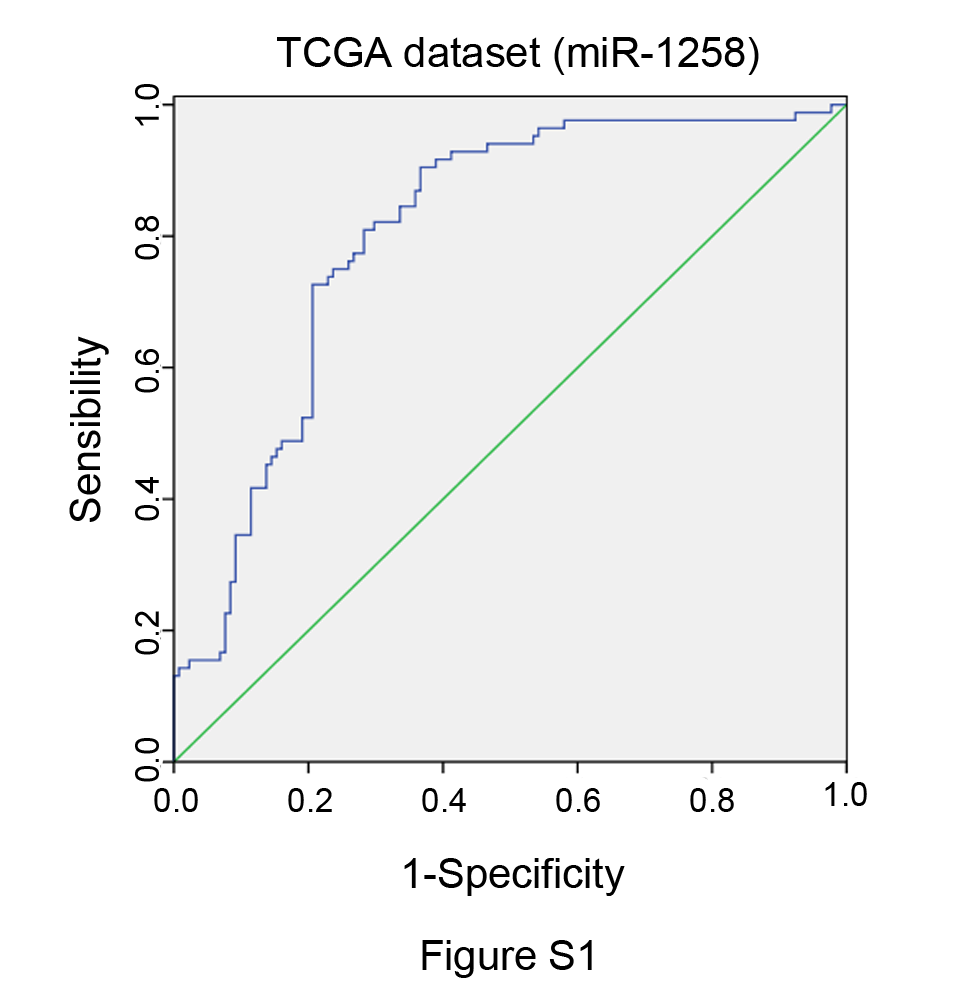

Supplement: Supplementary file 1 — Figure S1 [file 41388_2020_1307_MOESM1_ESM.tif]

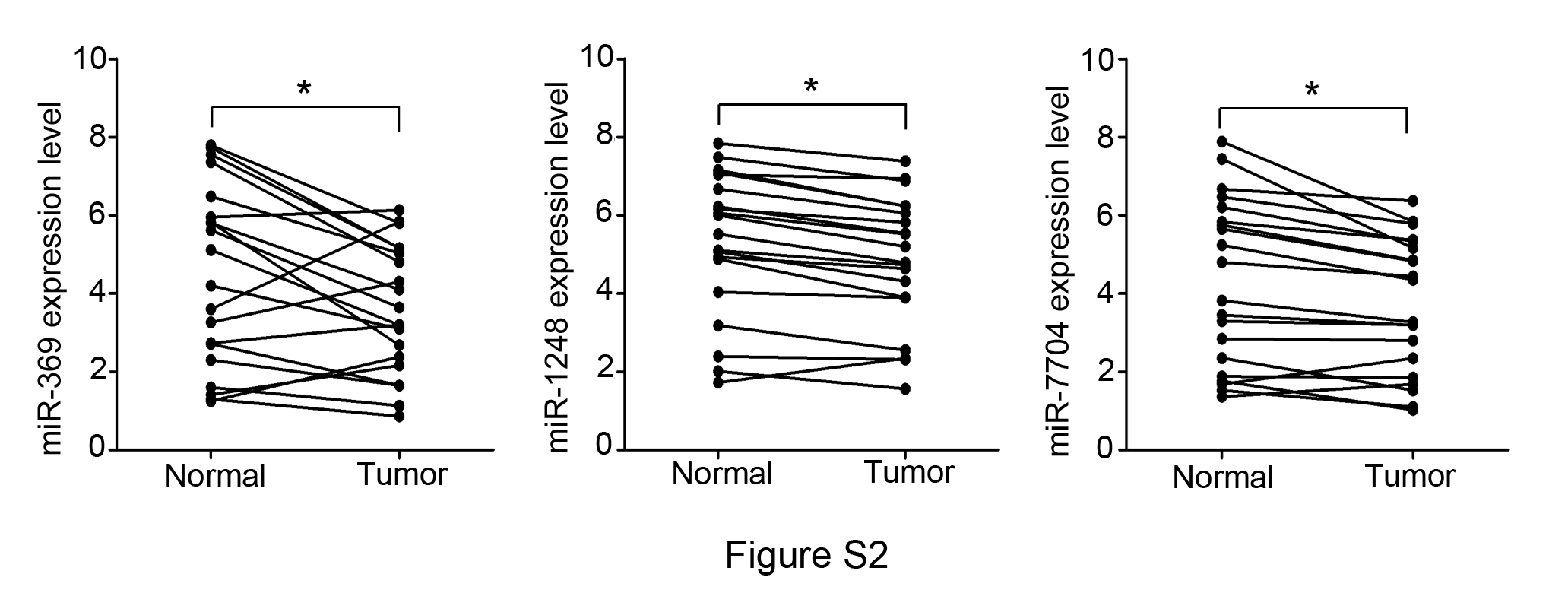

Supplement: Supplementary file 2 — Figure S2 [file 41388_2020_1307_MOESM2_ESM.tif]

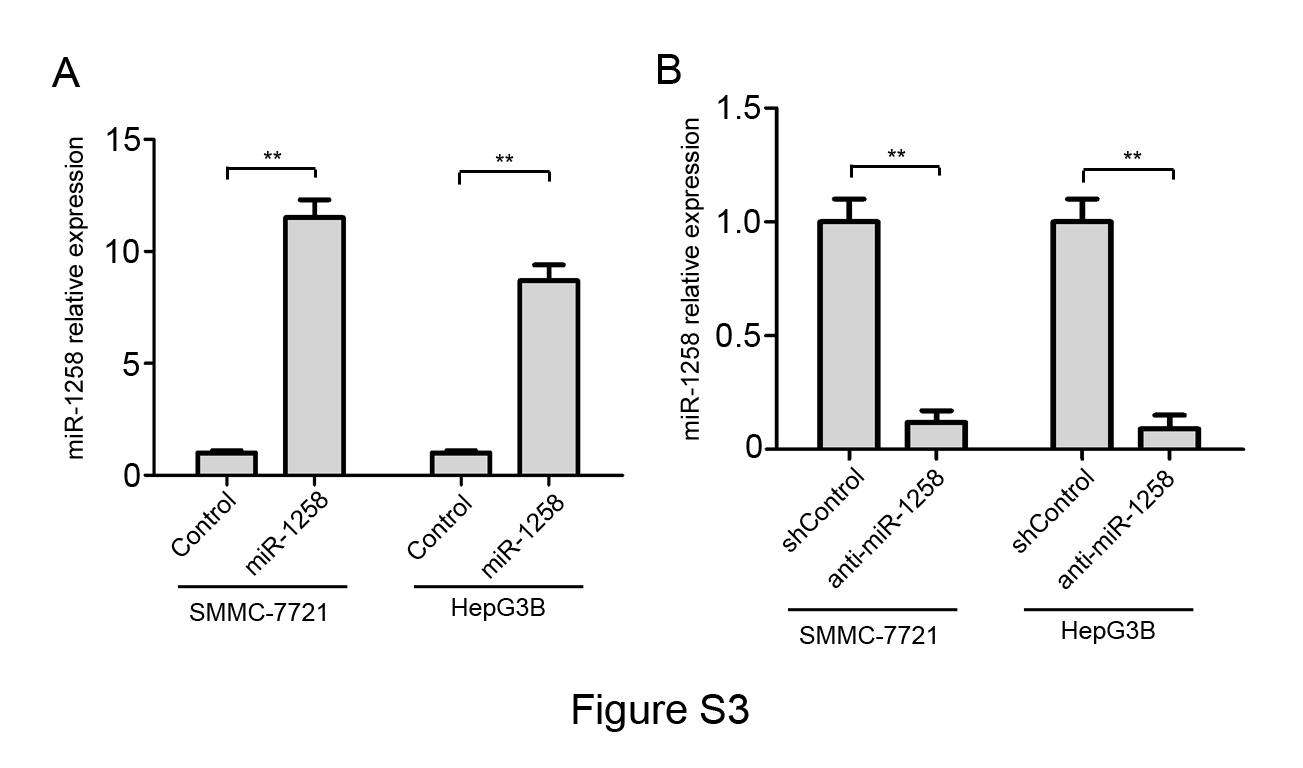

Supplement: Supplementary file 3 — Figure S3 [file 41388_2020_1307_MOESM3_ESM.tif]

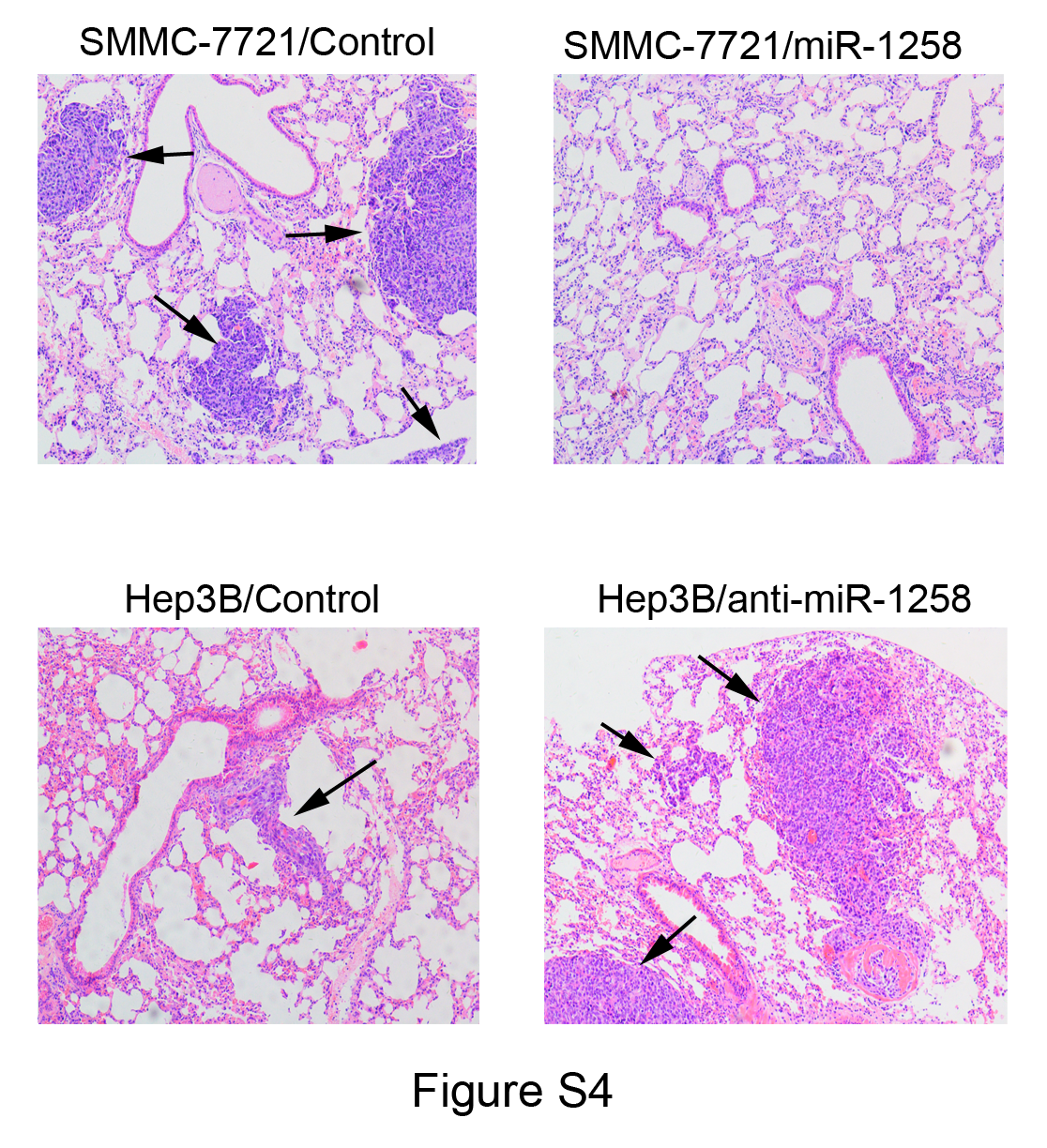

Supplement: Supplementary file 4 — Figure S4 [file 41388_2020_1307_MOESM4_ESM.tif]

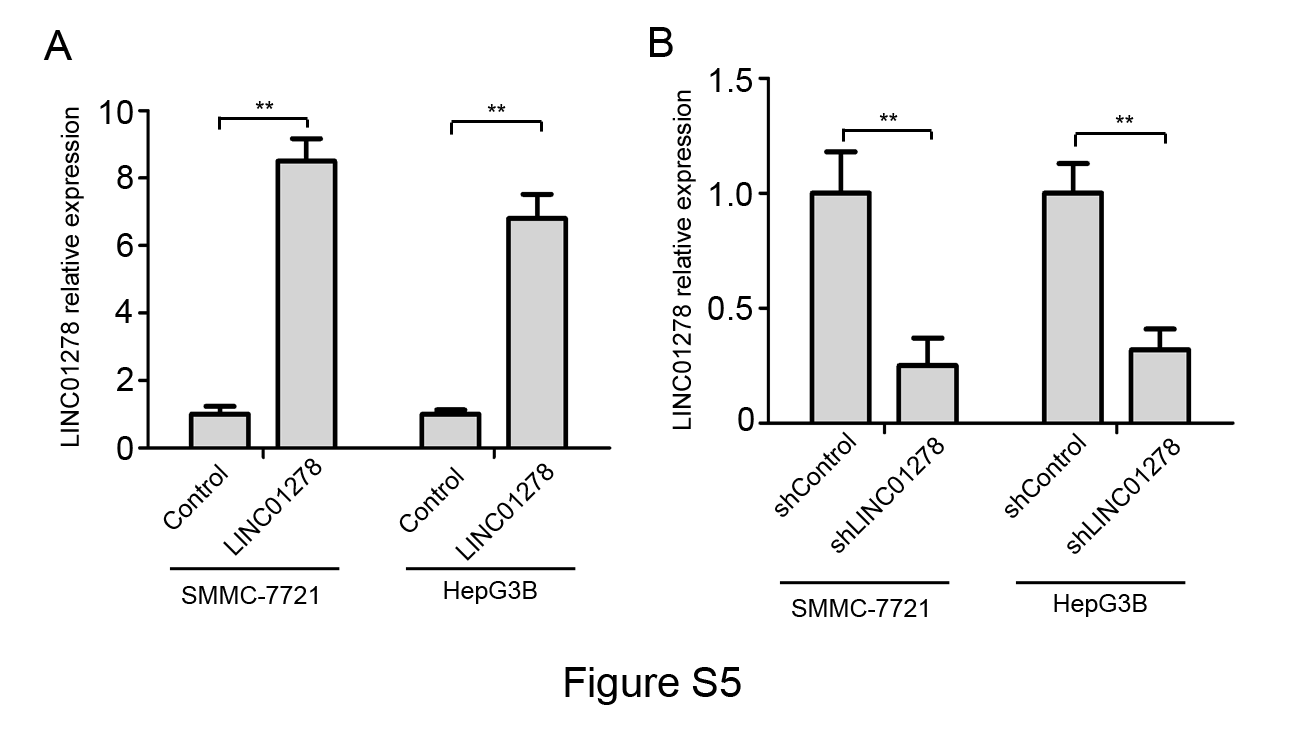

Supplement: Supplementary file 5 — Figure S5 [file 41388_2020_1307_MOESM5_ESM.tif]

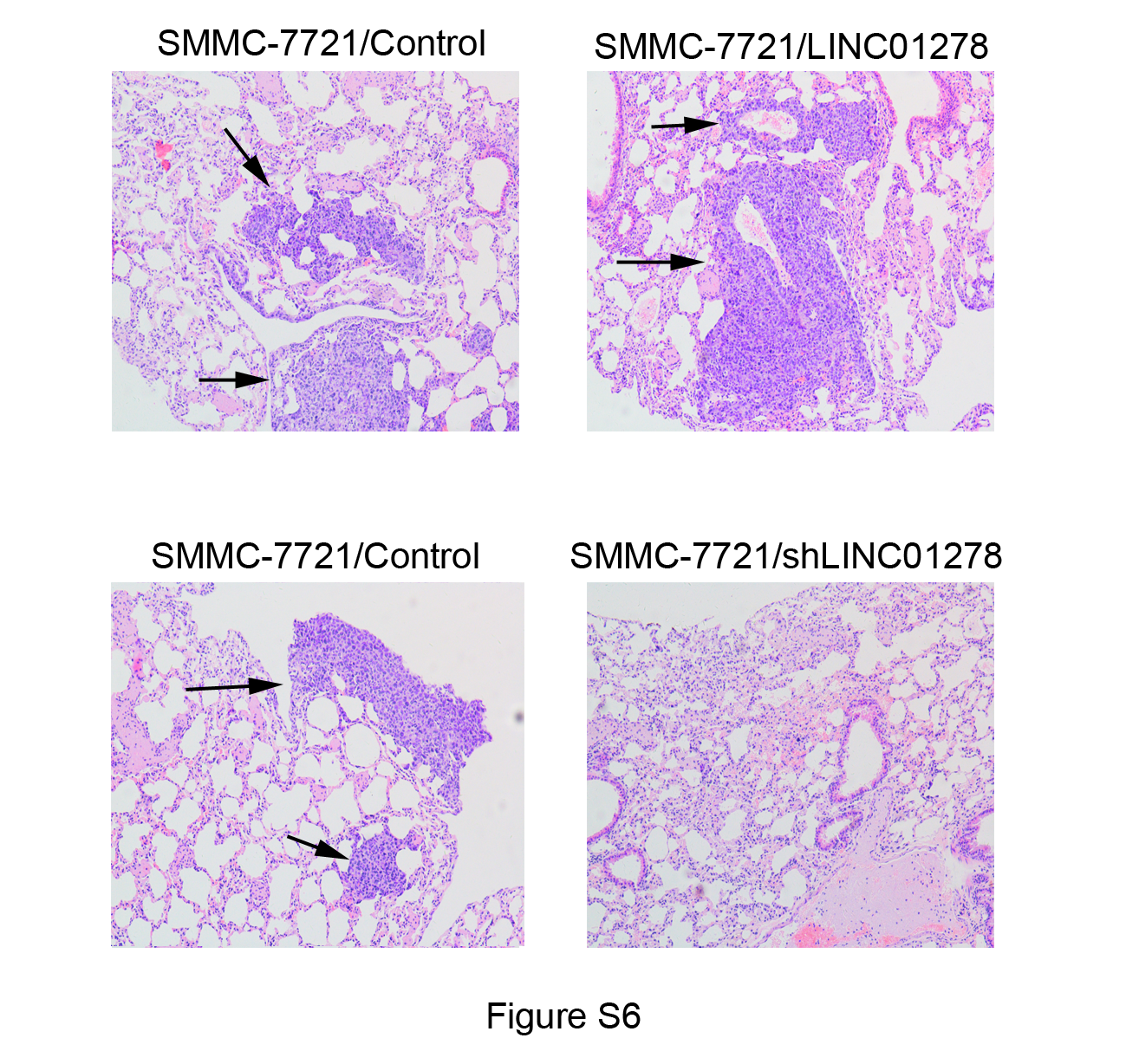

Supplement: Supplementary file 6 — Figure S6 [file 41388_2020_1307_MOESM6_ESM.tif]

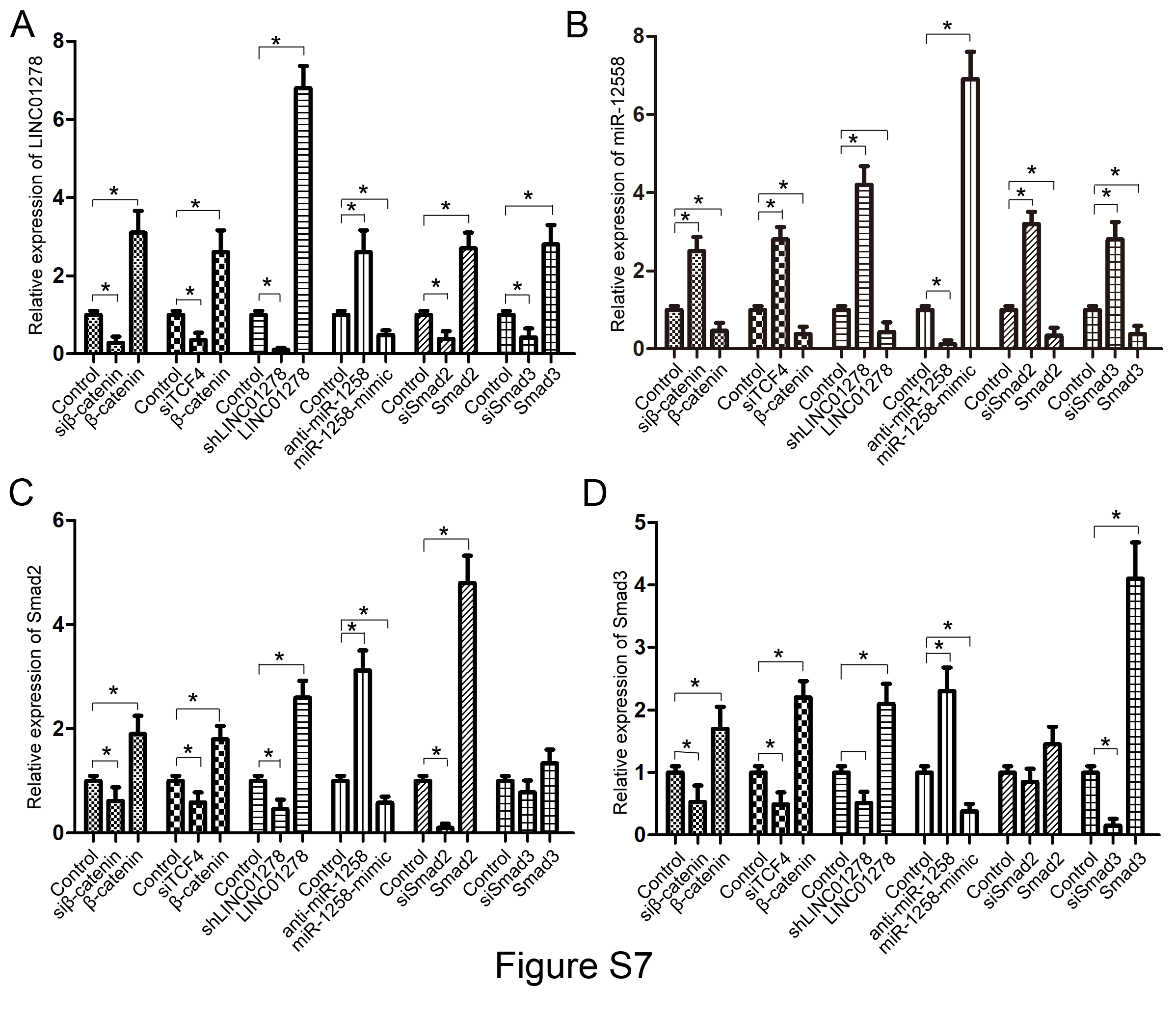

Supplement: Supplementary file 7 — Figure S7 [file 41388_2020_1307_MOESM7_ESM.tif]

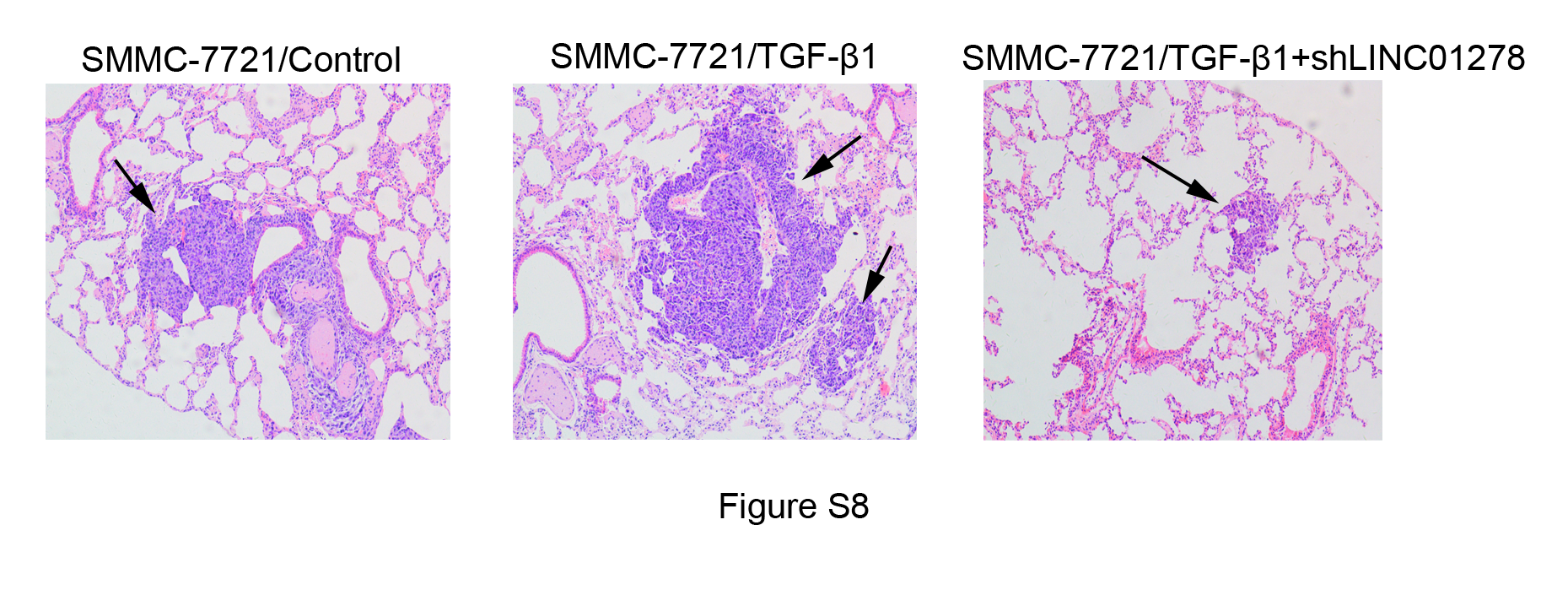

Supplement: Supplementary file 8 — Figure S8 [file 41388_2020_1307_MOESM8_ESM.tif]
