# Supplementary material for: The β-catenin/TCF-4-LINC01278-miR-1258-Smad2/3 axis promotes hepatocellular carcinoma metastasis
Source: Oncogene. 2020 May 5;39(23):4538–50. doi: 10.1038/s41388-020-1307-3 (PMC7269911; doi:10.1038/s41388-020-1307-3)
Supplement: Supplementary file 9 — Table S1 [file 41388_2020_1307_MOESM9_ESM.docx]

Table S1. The clinicopathologic characteristics of 117 early stage hepatocellular carcinoma patients stratified by low *vs.* high expression of miR-1258 and LINC01278.

| Variable | All cases | miR-1258 expression | | P value | LINC01278 expression | | P value |
| --- | --- | --- | --- | --- | --- | --- | --- |
|  |  | Low expression | High expression |  | Low expression | High expression |  |
| Age (years) |  |  |  |  |  |  |  |
| ≥48 | 58 | 27 | 31 | 0.649 | 31 | 27 | 0.642 |
| <48 | 59 | 25 | 34 |  | 29 | 30 |  |
| Sex |  |  |  |  |  |  |  |
| Male | 109 | 47 | 62 | 0.287 | 57 | 52 | 0.419 |
| Female | 8 | 5 | 3 |  | 3 | 5 |  |
| Hepatitis history |  |  |  |  |  |  |  |
| Yes | 96 | 42 | 54 | 0.747 | 49 | 47 | 0.911 |
| No | 21 | 10 | 11 |  | 11 | 10 |  |
| AFP (ng/mL) |  |  |  |  |  |  |  |
| ≤20 | 82 | 43 | 39 | 0.008 | 42 | 40 | 0.983 |
| >20 | 35 | 9 | 26 |  | 18 | 17 |  |
| Liver cirrhosis |  |  |  |  |  |  |  |
| Yes | 91 | 39 | 52 | 0.518 | 46 | 45 | 0.767 |
| No | 26 | 13 | 13 |  | 14 | 12 |  |
| Tumor size (cm) |  |  |  |  |  |  |  |
| ≤3 | 31 | 8 | 23 | 0.015 | 19 | 12 | 0.193 |
| >3 | 86 | 44 | 42 |  | 41 | 45 |  |
| Tumor multiplicity |  |  |  |  |  |  |  |
| Single | 96 | 35 | 61 | 0.000 | 54 | 42 | 0.022 |
| Multiple | 21 | 17 | 4 |  | 6 | 15 |  |
| Differentiation |  |  |  |  |  |  |  |
| Well-moderated | 87 | 38 | 49 | 0.776 | 44 | 43 | 0.794 |
| Poor-undifferentiated | 30 | 14 | 16 |  | 16 | 14 |  |
| Recurrence |  |  |  |  |  |  |  |
| No | 58 | 21 | 37 | 0.075 | 35 | 23 | 0.052 |
| Yes | 59 | 31 | 28 |  | 25 | 34 |  |

E-HCC: early-stage hepatocellular carcinoma; AFP: alpha fetoprotein; P value was calculated by Chi-square test.
