# Supplementary material for: The β-catenin/TCF-4-LINC01278-miR-1258-Smad2/3 axis promotes hepatocellular carcinoma metastasis
Source: Oncogene. 2020 May 5;39(23):4538–50. doi: 10.1038/s41388-020-1307-3 (PMC7269911; doi:10.1038/s41388-020-1307-3)
Supplement: Supplementary file 10 — Table S2 [file 41388_2020_1307_MOESM10_ESM.docx]

Table S2. Computationally predicted 10 lncRNAs capable of regulating miR-1258 in HepG2 cells and liver tissues.

| Gene | Transcript ID | Gene ID | Predict score |
| --- | --- | --- | --- |
| *KCNQ1OT1* | ENST00000597346 | ENSG00000269821 | 0.995 |
| *XLOC_004700* | TCONS_00010232 | XLOC_004700 | 0.970 |
| *XLOC_010133* | TCONS_00020848 | XLOC_010133 | 0.961 |
| *NFYC-AS1* | ENST00000606277 | ENSG00000272145 | 0.948 |
| *XLOC_000595* | TCONS_00001315 | XLOC_000595 | 0.947 |
| *RP3-393E18.2* | ENST00000430078 | ENSG00000237927 | 0.917 |
| *RP11-473M20.9* | ENST00000577123 | ENSG0000026237 | 0.915 |
| *LINC01278* | ENST00000608623 | ENSG00000235437 | 0.911 |
| *RP11-44F14.8* | ENST00000571340 | ENSG00000262714 | 0.902 |
| *RP11-705C15.3* | ENST00000537616 | ENSG00000257027 | 0.892 |
