# Supplementary material for: The β-catenin/TCF-4-LINC01278-miR-1258-Smad2/3 axis promotes hepatocellular carcinoma metastasis
Source: Oncogene. 2020 May 5;39(23):4538–50. doi: 10.1038/s41388-020-1307-3 (PMC7269911; doi:10.1038/s41388-020-1307-3)
Supplement: Supplementary file 11 — Table S3 [file 41388_2020_1307_MOESM11_ESM.docx]

Table S3. The sequence of primers.

| Gene name | Sequence |
| --- | --- |
| TCF-4 siRNA | SS sequence 5’-GGAGGUACAGACAAAGAAAGU-3’ |
|  | AS sequence 5’- UUUCUUUGUCUGUACCUCCAU-3’ |
| shLINC01278 | SS sequence 5’-GAAAGAGACTGTAGAGAAA-3’ |
|  | AS sequence 5’- TTTCTCTACAGTCTCTTTC-3’ |
| Smad-2 | Forward: 5’- TCCATCTTGCCATTCAC-3’ |
|  | Reverse: 5’-TTCTTCCTGCCCATTCT-3’ |
| Smad-3 | Forward: 5’- CGTGCGGCTCTACTACAT-3’ |
|  | Reverse: 5’- GGTTGCATCCTGGTGGG-3’ |
| LINC01278 | Forward: 5’- CCTGGTGTGCTGGCATCAAGTA-3’ |
|  | Reverse: 5’- TCTCCACTTCGCCACGGTCT-3’ |
