# Supplementary material for: The β-catenin/TCF-4-LINC01278-miR-1258-Smad2/3 axis promotes hepatocellular carcinoma metastasis
Source: Oncogene. 2020 May 5;39(23):4538–50. doi: 10.1038/s41388-020-1307-3 (PMC7269911; doi:10.1038/s41388-020-1307-3)
Supplement: Supplementary file 12 — Supplementary Figure Legends [file 41388_2020_1307_MOESM12_ESM.docx]

**Supplementary Figure legends**

**Figure S1. Receiver operating curve (ROC) analysis was used to determine the optimal cutoff value of miR-1258.**

**Figure S2. The expression levels of miR-369, miR-1248, miR-7704 were measured in 20 paired HCC tissues with adjacent normal tissues from SYSUCC dataset.**

**Figure S3. The expression of miR-1258 transfected by miR-1258 expression vector and anti-miR-1258 vector in HCC cells.**

**Figure S4. The number of metastatic lung nodules in miR-1258 overexpressing and anti-miR-1258 group.**

**Figure S5. The expression of LINC01278 transfected by LINC01278 expression vector and shLINC01278 vector in HCC cells.**

**Figure S6. The number of metastatic lung nodules in LINC01278 overexpressing and shLINC01278 group.**

**Figure S7. The mRNA levels of LINC01278, miR-1258, Smad2/3 by suppressing/elevating the expression of β-catenin, TCF-4, LINC01278, miR-1258, and Smad2/3 in HCC cells. A.** The relative expression of LINC01278. B**.** The relative expression of miR-1258. **C.** The relative expression of Smad2. **D.** The relative expression of Smad3. siβ-catenin, HCC cells were transfected by siRNA target β-catenin. β-catenin, HCC cells were transfected by β-catenin expression vector. siTCF4, HCC cells were transfected by siRNA target TCF-4. TCF-4, HCC cells were transfected by TCF-4 expression vector. shLINC01278, HCC cells were transfected by shRNA target LINC01278. LINC01278, ectopic LINC01278 expression in HCC cells. miR-1258, HCC cells were transfected by miR-1258 expression vector. Anti-miR-1258, HCC cells were transfected by miR-1258 antisense plasmid. siSmad2, HCC cells were transfected by siRNA target Smad2. Smad2, HCC cells were transfected by Smad2 expression vector. siSmad3, HCC cells were transfected by siRNA target Smad3. Smad3, HCC cells were transfected by Smad3 expression vector.*, *P*<0.05.

**Figure S8. The number of metastatic lung nodules in TGF-β1 and TGF-β1+shLINC01278 group.**
